# P019. Transcutaneous supraorbital neurostimulation in “*de novo*” patients with migraine without aura: the first Italian experience

**DOI:** 10.1186/1129-2377-16-S1-A136

**Published:** 2015-09-28

**Authors:** Antonio Russo, Francesca Conte, Laura Marcuccio, Alfonso Giordano, Gioacchino Tedeschi, Alessandro Tessitore

**Affiliations:** Department of Medical, Surgical, Neurological, Metabolic and Aging Sciences, Second University of Naples, Naples, Italy; MRI Research Center SUN-FISM, Second University of Naples, Naples, Italy; Institute for Diagnosis and Care “Hermitage Capodimonte”, Naples, Italy

## Background

Pharmacological anti-migraine preventive therapies are widely used to reduce the impact of migraine on quality of life; nevertheless, they may exhibit incomplete efficacy and significant side effects. Transcutaneous supraorbital neurostimulation (tSNS) has been recently proposed for the treatment of migraine. Moreover, tSNS has been recently found superior to sham stimulation for episodic migraine prevention in a randomized trial [1].

## Objective

To evaluate both the safety and efficacy of a brief period of tSNS in a group of patients with migraine without aura (MwoA). To this end, we used a tSNS medical device, called Cefaly® (CEFALY Technology, Herstal, Belgium), approved for use in migraine prevention by the Food and Drug Administration (FDA) and for sale in Europe.

## Methods

We enrolled 24 consecutive patients with MwoA experiencing a low frequency of attacks (≤5 attacks/month), whom had never taken migraine preventive drugs in the course of their life. Patients performed a daily supraorbital high frequency tSNS, for 20 minutes, for two months. Primary outcome measures were the reduction in migraine attacks and migraine days per month (p < 0.05). Secondary outcome measures were the reduction of headache severity during migraine attacks (by means of visual analogic scale) and HIT-6 (Headache Impact Test) rating, as well as in monthly intake of rescue medication (p < 0.05). Finally, compliance, treatment satisfaction, and potential adverse effects related to tSNS were evaluated.

## Results

Between run-in and second month of tSNS treatment, both primary and secondary endpoints were met. Indeed, we observed a statistically significant decrease in the frequency of migraine attacks (p < 0.001) and migraine days (p < 0.001) per month, as well as a reduction in average pain intensity during migraine attacks (p = 0.002), HIT-6 rating (p < 0.001), and intake of rescue medication (p < 0.001). All patients showed good compliance levels and no relevant adverse events occurred during the tSNS period.

## Conclusions

In patients with MwoA experiencing a low frequency of attacks, significant improvements in multiple migraine severity parameters was observed following a brief period of high frequency tSNS [2]. Therefore, tSNS may be considered a valid option for the preventive treatment of migraine attacks in patients who cannot or are not willing to take daily medications, or in which low migraine frequency and/or intensity would not require pharmacological preventive therapies.

Written informed consent to publish was obtained from the patient(s).
